# Familial Translocation t(2;4) (q37.3;p16.3), Resulting in a Partial Trisomy of 2q (or 4p) and a Partial Monosomy of 4p (or 2q), Causes Dysplasia

**DOI:** 10.3389/fgene.2021.741607

**Published:** 2021-11-23

**Authors:** Jian Wang, Shiyuan Zhou, Fei He, Xuelian Zhang, Jianqi Lu, Jian Zhang, Feng Zhang, Xiangmin Xu, Fang Yang, Fu Xiong

**Affiliations:** ^1^ Department of Fetal Medicine and Prenatal Diagnosis, Zhujiang Hospital, Southern Medical University, Guangzhou, China; ^2^ Department of Medical Genetics, School of Basic Medical Sciences, Southern Medical University, Guangzhou, China; ^3^ Henan Provincial Research Institute for Population and Family Planning Zhengzhou China, Zhengzhou, China; ^4^ Obstetrics and Gynecology Hospital, NHC Key Laboratory of Reproduction Regulation (Shanghai Institute of Planned Parenthood Research), State Key Laboratory of Genetic Engineering at School of Life Sciences, Fudan University, Shanghai, China; ^5^ Guangdong Provincial Key Laboratory of Single Cell Technology and Application, Guangzhou, China

**Keywords:** balanced translocation of chromosomes, 4p16.3, wolf-hirschhorn syndrome, 4p16.3 microduplication syndrome, ID/DD

## Abstract

**Background:** Wolf-Hirschhorn syndrome, a well-known contiguous microdeletion syndrome, is caused by deletions on chromosome 4p. While the clinical symptoms and the critical region for this disorder have been identified based on genotype-phenotype correlations, duplications in this region have been infrequently reported.

**Conclusion:** Our case report shows that both deletions and duplications of the Wolf-Hirshhorn critical region cause intellectual disability/developmental delay and multiple congenital anomalies.

**Case presentation:** We report on a family presenting with a set of dysmorphic facial features, attention deficit hyperactivity disorders, learning difficulties, speech and cognitive delays, overgrowth/developmental delay, and musculoskeletal anomalies. Through karyotyping, chromosomal microarray, and PCR analyses, it was found that patients in this family had translocations on chromosomes 2q37 and 4p16. Patients with 2q duplications and 4p deletions showed clinical phenotypes typical of WHS syndrome. Family members with 2q deletions and 4p duplications similarly manifested distinct clinical phenotypes.

## Background

Intellectual disability/developmental delay (ID/DD) is a common neuropsychiatric disorder with a complex etiology that includes external environmental factors and inherent genetic factors (Schalock et al., 2007). About 66.7% of ID is due to genetic factors. These can include mono- or polygenic diseases, abnormal gene copy numbers or chromosome counts, or structural abnormalities (Moeschler, 2008). Copy number variation (CNV) refers to the insertion, deletion, or amplification of DNA fragments from 1 kb to several Mb in size with resultant complex chromosomal structural variations when compared to reference sequences in the genome. Studies have shown that CNVs are found in 10–15% of patients with ID accompanied by congenital abnormalities (Kirov, 2015).

Wolf-Hirschhorn syndrome (WHS) syndrome is a classic example of a CNV-associated genetic condition. WHS is caused by differently sized deletions of the 4p chromosomal region. Notably, the deletion of the *WHSC1* gene is considered to be the cause of the facial appearance in WHS patients (Andersen et al., 2014). The core characteristics of WHS include delayed prenatal and postnatal growth, craniofacial hypoplasia, delayed development, and epilepsy (Bergemann et al., 2005; Engbers et al., 2009; Nevado et al., 2020). Typical craniofacial features include prominent glabellas, microcephaly, a “Greek warrior helmet appearance” of the nose, widely spaced and prominent eyes, a short philtrum, a broad nasal tip, and downturned corners of the mouth (Titomanlio et al., 2004). Identification of this condition strongly depends on recognition of the facial gestalt.

In this study, we report on a family in which several members have CNVs occurring on chromosomes two and 4. Their clinical phenotypes include ID, abnormal development, slow reactions, and abnormal skeletal development. Some patients presented with poor suction after birth. Therefore, a detailed survey of the family members was conducted, with karyotype analysis and CNV testing confirming the diagnoses.

## Materials and Methods

### Patients

A detailed survey was conducted of all family members. The data were recorded and sorted according to the phenotypes of each member. Representative photos of the phenotype were obtained from some family members. Peripheral venous blood for DNA extraction was collected into evacuated EDTA tubes from most members of the family. Genomic DNA was prepared from peripheral blood leukocytes. This study was approved by the Zhujiang Hospital Ethics Committee, an affiliate of the Southern Medical University (Guangdong, China).

### Karyotyping

Karyotyping was performed on phytohemagglutinin-stimulated peripheral blood lymphocytes according to standard clinical procedures. Karyotypes of the family members were analyzed using G-banding chromosome analysis (Zhang et al., 2020). The karyotypes were described according to the International System for Human Cytogenomic Nomenclature standards (ISCN 2016). Partial cell suspensions were stored at −20°C.

### Chromosomal Microarray Analysis

To further understand if minor deletions were present on the chromosomes, comparative genomic hybridization experiments (array CGH) were performed on five samples using the Affymetrix Cytoscan® 750 k (Affymetrix, Santa Clara, CA, United States). This platform includes 200,000 SNP markers and 500,000 copy number variation (CNV) markers that are distributed across the human genome at an average density of about 1 marker/4 kb (Zhang et al., 2020). The arrays were strictly run according to the manufacturer’s protocol and the data were visualized and analyzed using the Chromosome Analysis Suite software package (Affymetrix). The February 2009 human reference sequence (GRCh37/Hg19, http://genome.ucsc.edu/) was used as the reference sequence.

### PCR Amplification

Primers were designed and the duplication or deletion of chromosome fragments was verified in different patients through PCR. To verify the balanced translocation through PCR analysis, two sets of primers were designed for the chromosome where the translocation occurred. PCR was performed with specific oligonucleotide primers as follows: chromosome 4 binding primer F: 5′- GTC​CCG​GTC​CAT​AAC​GCT​TGC -3′ and R: 5′- GGA​AGT​TAT​GTG​TAC​CGG​AT -3’ (amplified fragment length of 420bp); chromosome two binding primer F: 5′- GAT​CAT​TAC​CGA​GTC​TTT​CTG -3′ and R: 5′- CGT​CTG​GGT​TGC​CAC​AGA​CGA -3’ (amplified fragment length of 550 bp). The PCR products were analyzed by electrophoresis in 2% agarose gel, followed by ethidium-bromide staining.

## Results

### Case Presentation

**FIGURE 1:**
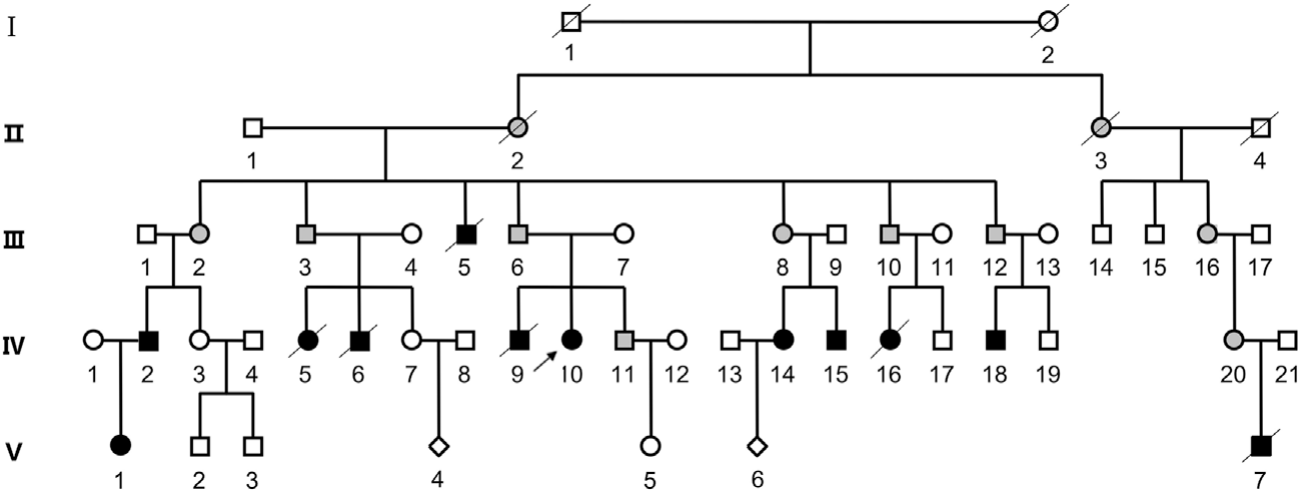
Pedigree of the patient’s family. Squares represent male family members, while circles represent female family members. The diamond symbol indicates unknown gender. Slash indicates that the family member is deceased. Black symbols represent individuals with ID/DD, and blank symbols represent unaffected family members. The gray filled symbols indicates balanced translocation carriers.

The proband was a 25-year-old woman who went to the hospital for medical examination due to ID. No abnormalities were found on electroencephalogram tests. It was learned through interviews that this phenomenon also existed in some of the family members of this woman’s family. Among the family members, three had similar physical characteristics as the proband. These included tall, overweight, signs of dementia on the face, duck steps, and ID. In contrast, seven other family members had short, underweight statures, stunted growth, and were unable to restrict their behavior. The pedigree of this family is shown in Figure 1, while the detailed phenotypic records of family members are shown in Table 1. The differences among healthy, deletion, duplication, and translocation patients are shown in Table 2.

**TABLE 1:** Details of the family members.

Clinical phenotype at diagnosis	Gender and age	Height (cm)	Weight (kg)	Development	Nerve state	Language skills	Nutritional status	Muscles and bones	Craniofacial features	Activity and gait	Other
II-1	M,85	153	61	good	normal	After tracheotomy	good	normal	normal	normal	**\**
III-3	M,59	168	74	good	normal	normal	good	normal	normal	normal	**\**
III-4	F,60	153	54.5	good	normal	normal	good	normal	normal	normal	**\**
III-5	M	170	**\**	**\**	Congenital dementia	Can communicate with others simply, but often give an irrelevant answer	**\**	**\**	**\**	Cannot restrain one’s behavior	died of electric shock at the age of 44. Can take care of himself, can do simple housework, can’t read, can’t recognize numbers, and has no initiative
III-6	M,50	165	65	good	normal	normal	good	normal	normal	normal	**\**
III-7	F,50	158	62	good	normal	normal	good	normal	normal	normal	**\**
III-8	F,48	150	61	good	normal	normal	good	normal	normal	normal	**\**
III-9	M,46	172	132	good	normal	normal	good	normal	normal	normal	**\**
III-10	M	162		good	normal	normal	good	normal	normal	normal	**\**
III-11	F,43	162	56.5	good	normal	normal	good	Mild muscular atrophy in the left lower limb	normal	normal	The right eyeball is staring to the right slightly, with normal activities; the left limb is not flexible, and the calculation ability is poor
III-12	M,40	170	95	good	normal	normal	good	normal	normal	normal	**\**
IV-2	M,31	170	80	**\**	Slow response	**\**	**\**	**\**	Dull face, drooping upper left eyelid	**\**	3.3 kg at birth
IV-5	F	**\**	**\**	Stunting	**\**	**\**	Malnutrition	Weak sucking power	**\**	**\**	Weak sucking power, vomiting after eating, stunted physical development, discharge diagnosed as malnutrition.5–6 months after birth, convulsions occur, and the seizures gradually increase from small to large, Until the major attack, died of convulsions before the age of two
IV-6	M	**\**	**\**	Stunting	**\**	**\**	Malnutrition	Weak sucking power	**\**	**\**	Weak sucking power, vomiting after eating, stunted physical development, discharge diagnosed as malnutrition. Convulsions occurred 5–6 months after birth, and the seizures gradually increased from childhood to major seizures and died of convulsions before the age of two
IV-7	F,24	156	49	good	normal	normal	good	normal	normal	normal	Intrauterine pregnancy 40 + days (V4)
IV-9	M	**\**	**\**	**\**	**\**	**\**	Severe malnutrition	**\**	**\**	**\**	Low birth weight, about 2.5 kg, no sucking power, died 1 month after birth
IV-10	F,25	163	90	good	Slow response	Can communicate with others, but often give an irrelevant answer	good	Flexion of the left knuckle of the two knuckles, no major knuckle of the hand, developmental defects of the index finger and ring finger of both hands; bilateral flat feet, large feet (wearing size 44 shoes)	Dementia face, dull expression, slightly drooping left eyelid	Walk like a duck	No abnormalities in EEG examination and low intelligence test value, no literacy, no numeracy
IV-11	M,21	172	63.5	good	normal	normal	good	normal	normal	normal	Congenital heart disease, diagnosed as ventricular septal defect, surgery at 7 years old, everything is normal after
IV-14	F,24	157	95	good	Slow response	Not very fluent in speaking, can communicate with others, but often give an irrelevant answer	good	Poor thumb development in both hands, shortened joint deformities; collapse of the bridge of the nose; flat feet on both sides	Dementia face, Stiff expression; small eyes, drooping upper left eyelid, full moon face	Walk like a duck	7 months pregnant
IV-15	M,17	130	30	Stunting	Slow response and naive behavior	Weak tongue, dysarthria, Slurred speech	Congenital malnutrition	Limited flexion and extension of the little fingers of both hands	Childish face	active, cannot restrain one’s behavior	2 kg weight at birth, sucking power is acceptable, but small appetite: no literacy, no numeracy
IV-16	**\**	**\**	**\**	**\**	**\**	**\**	**\**	**\**	**\**	**\**	low weight at birth, about 2 kg, no suction, and died about a month after birth
IV-17	M,19	160	52.5	good	normal	normal	good	normal	normal	normal	Poor computing power
IV-18	M,13	110	20	Stunting	Slow response and naive behavior	Impossibility of tongue extension, dysarthria, slurred speech, often give an irrelevant answer	Malnutrition	lower than his peers, Flexion of both thumbs	Horror face, Head circumference 48 cm	active, cannot restrain one’s behavior	weighs 2 kg at birth, has a weak sucking power, and eats less. No secondary sexual characteristics, no literacy, no numeracy
IV-19	M,8	128	24	good	normal	normal	good	normal	normal	normal	
V-1	F,2	**\**	**\**	**\**	Slow response	Slurred speech	**\**	**\**	**\**	**\**	Suspected mild intellectual disability
V-5	F,2.5		14.6	good	normal	normal	good	normal	normal	normal	**\**
V-7	**\**	**\**	87	**\**	**\**	**\**	**\**	**\**	**\**	**\**	Death at 12 years old, appearance and clinical manifestations are the same as IV18

**TABLE 2:** Differences among healthy, deletion, duplication, and translocation patients.

Patient	Rearrangement	Development	Nerve state	Language skills	Nutritional status	Muscles and bones	Craniofacial features	Activity and gait	Other
III-7	46, (XX)	good	normal	normal	good	normal	normal	normal	**\**
IV-10	der (2) t (2; 4) (q37.3; p16.3)	good	Slow response	Can communicate with others, but often give an irrelevant answer	good	Flexion of the left knuckle of the two knuckles, no major knuckle of the hand, developmental defects of the index finger and ring finger of both hands; bilateral flat feet, large feet (wearing size 44 shoes)	Dementia face, dull expression, slightly drooping left eyelid	Walk like a duck	No abnormalities in EEG examination and low intelligence test value, no literacy, no numeracy
IV-14		good	Slow response	Not very fluent in speaking, can communicate with others, but often give an irrelevant answer	good	Poor thumb development in both hands, shortened joint deformities; collapse of the bridge of the nose; flat feet on both sides	Dementia face, Stiff expression; small eyes, drooping upper left eyelid, full moon face	Walk like a duck	7 months pregnant
IV-15	der (4) t (2; 4) (q37.3; p16.3)	Stunting	Slow response and naive behavior	Weak tongue, dysarthria, Slurred speech	Congenital malnutrition	Limited flexion and extension of the little fingers of both hands	Childish face	active, cannot restrain one’s behavior	2 kg weight at birth, sucking power is acceptable, but small appetite; no literacy, no numeracy
IV-18		Stunting	Slow response and naive behavior	Impossibility of tongue extension, dysarthria, slurred speech, often give an irrelevant answer	Malnutrition	lower than his peers, Flexion of both thumbs	Horror face, Head circumference 48 cm	active, cannot restrain one’s behavior	weighs 2 kg at birth, has a weak sucking power, and eats less. No secondary sexual characteristics, no literacy, no numeracy
III-3	t (2; 4) (q37.3; p16.3)	good	normal	normal	good	normal	normal	normal	**\**
III-12		good	normal	normal	good	normal	normal	normal	**\**
IV-11		good	normal	Z	good	normal	normal	normal	Congenital heart disease, diagnosed as ventricular septal defect, surgery at 7 years old, everything is normal after

**FIGURE 2:**
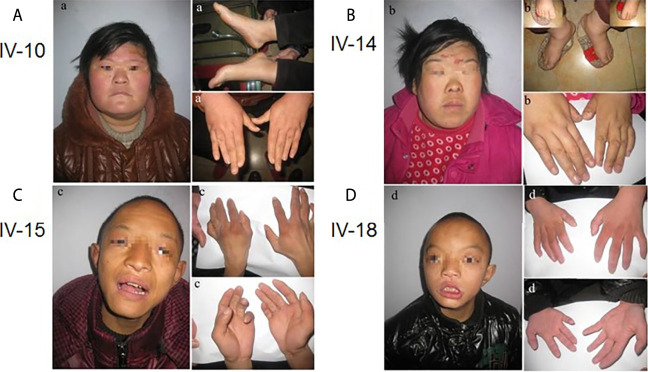
Facial features, hand and foot characteristics of patients with more obvious symptoms in the family.

Among the surviving family members, the clinical details of the patients with more obvious symptoms are as follows:

IV-10: This 25-year-old female measured 163 cm in height, weighed 90 kg (overweight), and presented with small eyes, a drooping upper left eyelid, a full moon face, wide palms, lack of joints between the ring finger and metacarpal, a short ring finger, and large feet (wearing size 44 shoes on the European scale). Born at full term (40 weeks), she weighed 5 kg at birth with good post-natal feeding. Facial reaction dementia was noted 6–7 months after birth, but no abnormalities were found in the hospital following an EEG examination and other tests. Her intelligence test value was low. While she can take care of herself and communicate with people, she often gives irrelevant answers to questions. She was illiterate and lacked numeracy skills. She walks like a duck (Figure 2A).

IV-14: This 24-year-old female measured 157 cm in height and weighed between 90 and 95 kg. She weighed 3.2 kg at birth. She is married and was currently pregnant *in utero* for 7 + months (V-6). Except for typical hand bones and joints, the remaining clinical manifestations were the same as IV-10 (Figure 2B).

IV-15: This 17-year-old male measured 130 cm in height and weighed 25 kg (underweight). Born at full-term (40 weeks), he weighed 4 kg at birth. His suction was diminished, he ate less, and he developed slowly. The hospital diagnosed the patient with congenital malnutrition. While this patient is active, he is not good at communicating with others, is unable to pronounce words clearly, and cannot restrict his behavior. He was illiterate and had no numeracy skills (Figure 2C).

IV-18: This 13-year-old male measured 110 cm in height, was underweight, had big eyes and double eyelids, and a pointed chin. He weighed 2 kg at birth, had poor suction, ate less frequently when compared to the norm, and was stunted in growth. No abnormalities were seen during the hospitalization. While this patient is active, he cannot restrain his own behavior, is unable to pronounce words clearly, is not good at communicating with others, and often provides irrelevant answers to questions. He had no literacy or numeracy skills. His body posture and clinical manifestations were the same as for patient IV-15 (Figure 2D).

### Karyotyping

In order to determine whether the patients of this family had intellectual disabilities due to chromosomal abnormalities, blood samples of some of the family members (patients and healthy control samples) were subjected to chromosome analysis. The results showed that the karyotypes of III-3, III-4, III-6, III-7, III-8, III-9, III-12, IV-7, IV-10, IV-11, IV-14, IV-15, IV-18, and IV-19 were normal.

### Chromosomal Microarray Analysis

**FIGURE 3:**
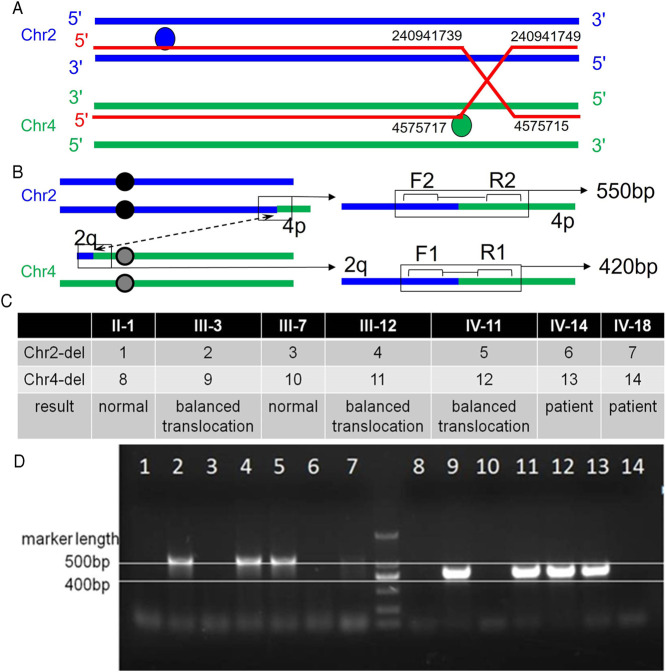
Primer position and agarose gel electrophoresis results. A: The breakpoint and binding position of the ectopic occurrence. Balanced translocation carriers have reciprocal and symmetric translocations, t (2; 4) (q37.1; p16.2). B: Primer position diagram. The forward and reverse primer binding positions of the first pair of primers are 2q and 4q respectively, that is, they can only bind to chromosome 4 with a 2q fragment. The second set of primers can also only bind to chromosome two and 4p fragments. C: Analysis of electrophoresis results. Bands are present in both primer pairs, indicating that the sample source represents a balanced translocation carrier. Neither band indicates that the sample source is normal. Only one pair of primers indicates that the sample source is that of a patient. D: Electrophoresis results.

To further understand if there were minor deletions on the chromosomes, comparative genomic hybridization experiments (array CGH) were performed on five samples. The proband (IV-10) and IV-14 were found to harbor the same CNVs, with a 2.2 Mb 2q terminal deletion on chromosome two and a 4.6 Mb 4p terminal duplication on chromosome 4. The latter included the entire 4p16.3 region and part of 4p16.2. However, IV-18, who has a different phenotype, was found to have the opposite CNVs with a 2.2 Mb 2q terminal duplication noted on chromosome two and a 4.6 Mb 4p terminal deletion on chromosome 4. Besides there being a difference in length of less than 10bp, the deletion and duplication positions were the same as IV-10 and IV-14. The CNVs of chromosomes two and 4 of the above-mentioned patients originated from the same balanced translocation (Figure 3A). In addition, IV-10 not only possessed a 2.2 Mb 2q terminal deletion on chromosome two and 4.6 Mb 4p terminal duplication on chromosome 4, but also a 411 kb Xp duplication on her X chromosome. Among the family members, III-6 and IV-11 are in good health without any evidence of dysplasia. Except for congenital heart disease found in IV-11, all remaining family members are healthy.

### Detection of Some Family Members by PCR Analysis

Based on the CGH results, it was speculated that a balanced translocation was present in the family members. To verify this, two sets of primers were designed for the chromosomes where the translocation occurred (Figure 3B). The forward and reverse primer binding positions of the first set of primers were for 2q and 4q respectively, that is, they could only bind to chromosome 4 with 2q fragments. The second set of primers were constructed in a similar way in that 4p fragments would bind to chromosome 2. The two sets of primers are used to amplify the patient’s DNA. Banding noted on gel electrophoresis confirmed the presence of the translocation, while an absence of bands indicated normal chromosomal structure. Combining the two sets of results indicated the presence or absence of the translocation of chromosomes 2 and 4 in family members (Figures 3C,D).

## Discussion

In this study, a large family with an inherited balanced translocation between chromosomes two and 4 is described. This translocation resulted in either a 4.6 Mb deletion at chromosomal position 4p16.3 and a 2.2 Mb duplication at chromosomal position 2q37.3, or a 4.6 Mb duplication at 4p16.3 and a 2.2 Mb deletion at 2q37.3 in the offspring. By combining the patients’ conditions, PCR amplification results, and CGH chip analysis results, a pedigree analysis could be performed for this family. Among the pedigrees developed from II-1 and II-2, it was noted that each family had an affected individual in the fourth generation, while balanced translocations were observed for family members III-3, III-12, and IV-11. It can be speculated that the offspring of II-1 and II-2 all possessed a balanced translocation. As their spouses demonstrated normal phenotypes, the fourth generation of patients and normal phenotypes appeared at the same time.

While the chromosomal phenotype of II-1 was completely normal, all offspring possessed balanced translocations. It is possible that germ cell mutations in II-2 resulted in homozygous translocations in the offspring. However, this hypothesis cannot explain the cause of the clinical presentation of III-5. As this patient had died, it was not possible to determine his chromosomal phenotype. It is therefore speculated that unknown, spontaneous changes occurred during the formation of gametes in both parents. Due to a lack of data, the family that extended from II-3 and II-4 could not be analyzed. It is theorized that it is possible that the clinical features of both III-16 and IV-20 are due to balanced translocations, and that patient V-7 harbors a dup2q + del4p variation due to having a clinical phenotype that is the same as patient IV-18. This scenario has a lower probability of being correct.

Based on the array CGH results, it is hypothesized that the cause of the disease in this family is mainly related to the deletion or duplication of 4p16.3. Microdeletions on the short arm of chromosome 4 are associated with WHS, which is a rare disease with an incidence of about 1/200 to 1/500,000. While the size of the deletion varies from person to person, studies have shown that larger deletions can lead to more severe ID and physical abnormalities (Akhtar, 2008; Dai et al., 2013; Battaglia et al., 2015). In this family, the 4p16.3 region of IV-18 was completely deleted. This included the deletion of the *WHSC1* gene. As the phenotype of this patient matched the core characteristics of WHS, it is believed that IV-18 suffers from WHS. The phenotypes of other affected family members may also be associated with WHS.

Similarly, duplications on chromosome 4 are likely to be pathogenic, with three cases having been reported to date (Hannes et al., 2010; Cyr et al., 2011; Palumbo et al., 2015). All three cases presented with the following clinical features: musculoskeletal abnormalities, psychomotor and speech delays, craniofacial abnormalities (frontal lobe protrusion, high forehead, short neck, hyperopia/blepharochalasis, epicanthal folds). These common features suggest that occult 4p16.3 replication leads to a novel recognizable microreplication syndrome; one patient was diagnosed with a form of giantism, while another patient was noted to present with overgrowth. In addition, a large study on 4p16.3 microdeletions and microduplications also provides evidence for 4p16.3 microduplication syndrome (Bi et al., 2016). The phenotypes of patients with duplicated 4p regions in this family, namely IV-10 and IV-14, are similar to those characteristics associated with 4p16.3 microduplication syndrome. SNP array analysis showed a 4p16.3 duplication of a maximum size of 413 kb, from nucleotide 1,390,388 to nucleotide 1,804,276, and a minimum duplication size of 393 kb, from nucleotide 1,405,662 to nucleotide 1,798,461. The repeated region contains the complete coding sequence of the 5′ ends of the *FAM53A*, *SLBP*, *TMEM129*, *TACC3*, and *FGFR3* genes. The *FAM53A*, *TACC3*, and *FGFR3* genes appear to play key roles in the etiology of the clinical phenotype (Palumbo et al., 2015). The deletions and duplications on 4p in this family include the entire 16.3 region. Therefore, it is believed that the cause of the illness in patients IV-10 and IV-14 is likely related to the duplication of 4p. Family members with phenotypes like that observed for patients IV-10 and IV-14 may similarly harbor this duplication.

The position of the deletions or duplications on chromosome two were found to be part of region 2q37.3. Deletions of this region have been the focus of studies targeting 2q37.3 deletion syndrome cases. This syndrome belongs to the chromosome 2q37 deletion disorder spectrum; some of its core phenotypes also manifest in this family. As for the duplication of 2q37.3, there is no definite report about the pathogenicity of this region, so the effect of it on patients is not clear. Duplications or deletions of 2q37.3 and their effects on human health therefore need further research.

Based on the analyses conducted in this study, we believe that the deletion or duplication of fragments on chromosome 4 resulted in patients with complex phenotypes within families. Deletions and duplications in the 4p16.3 region appeared to cause two reciprocal syndromes: WHS and 4p16.3 microduplication syndrome. In this report, we provide clinical and molecular evidence supporting the existence of 4p16.3 microduplication syndrome, expand the database related to both syndromes, and provide a new basis for the prenatal diagnosis of these two conditions.

## Data Availability

The original contributions presented in the study are included in the article/supplementary material, further inquiries can be directed to the corresponding author.
